# Glucose oxidase and metal catalysts combined tumor synergistic therapy: mechanism, advance and nanodelivery system

**DOI:** 10.1186/s12951-023-02158-w

**Published:** 2023-10-31

**Authors:** Yuhan Fu, Jialin Sun, Yanhong Wang, Weinan Li

**Affiliations:** 1 School of Pharmacy, Heilongjiang University of Chinese Medicine, Harbin, Heilongjiang Province China; 2 Key Laboratory of Basic and Application Research of Beiyao Ministry of Education, Heilongjiang University of Chinese Medicine, Harbin, Heilongjiang Province China; 3 Postdoctoral Research Station, Heilongjiang University of Chinese Medicine, Harbin, Heilongjiang Province China; 4 Biological Science and Technology Department, Heilongjiang Minzu College, Harbin, Heilongjiang Province China

**Keywords:** Synergistic therapy, Glucose oxidase, Metal catalysis, Cascade catalysis, Nanoparticles, Cancer therapy

## Abstract

Cancer has always posed a significant threat to human health, prompting extensive research into new treatment strategies due to the limitations of traditional therapies. Starvation therapy (ST) has garnered considerable attention by targeting the primary energy source, glucose, utilized by cancer cells for proliferation. Glucose oxidase (GOx), a catalyst facilitating glucose consumption, has emerged as a critical therapeutic agent for ST. However, mono ST alone struggles to completely suppress tumor growth, necessitating the development of synergistic therapy approaches. Metal catalysts possess enzyme-like functions and can serve as carriers, capable of combining with GOx to achieve diverse tumor treatments. However, ensuring enzyme activity preservation in normal tissue and activation specifically within tumors presents a crucial challenge. Nanodelivery systems offer the potential to enhance therapy effectiveness by improving the stability of therapeutic agents and enabling controlled release. This review primarily focuses on recent advances in the mechanism of GOx combined with metal catalysts for synergistic tumor therapy. Furthermore, it discusses various nanoparticles (NPs) constructs designed for synergistic therapy in different carrier categories. Finally, this review provides a summary of GOx-metal catalyst-based NPs (G-M) and offers insights into the challenges associated with G-M therapy, delivery design, and oxygen (O_2_) supply.

## Introduction

Cancer has emerged as a serious threat to human health and life [1]. The primary treatments for cancer include radiotherapy, chemotherapy (CT), and surgery [2]. Despite yielding positive clinical outcomes, concerns persist regarding the treatment process. For instance, radiotherapy and surgery can cause damage to healthy tissues, while tumor drug resistance to CT drugs diminishes treatment effectiveness [3, 4]. Consequently, there is an urgent need for a new treatment strategy to overcome these challenges. Tumor cells follow the “Warburg effect” in their glucose metabolism pathway, utilizing aerobic glycolysis to generate energy even in the presence of oxygen (O_2_) [5]. When ample glucose is available, glycolysis produces adenosine triphosphate (ATP) at a rate surpassing oxidative phosphorylation, thereby providing abundant energy for tumor growth. Thus, rapid glucose depletion to completely suppress the tumor presents a promising therapeutic strategy. Glucose oxidase (GOx), a natural oxidoreductase, possesses a specific binding site for D-glucose [6]. Under GOx catalysis, large quantities of O_2_ and glucose are swiftly consumed, leading to the production of hydrogen peroxide (H_2_O_2_) and D-gluconic acid. Due to its rapid glucose consumption ability, GOx has gained popularity as a therapeutic agent for tumor starvation therapy (ST) [7]. Notably, the tumor pH ranges between 5 and 7, with the highest activity of GOx observed at pH 6.5 [8]. Additionally, GOx operates optimally within the human body temperature range of 37 °C (between 30 °C and 60 °C), further enhancing its potential in cancer therapy.

However, the ST treatment course is constrained by insufficient O_2_ supply to the tumor. To address the limitation of O_2_ in ST, certain metal catalysts generate O_2_ through catalase-like (CAT-like) activity, maximizing the utility of GOx [9]. Moreover, some metal catalysts utilize the H_2_O_2_ produced by GOx to induce the Fenton reaction, enabling the combination of chemodynamic therapy (CDT) and ST [10]. Currently, based on the combination of GOx and metal catalysts, cancer therapy modalities primarily include CT, ST, CDT, photodynamic therapy (PDT), sonodynamic therapy (SDT), photothermal therapy (PTT), immunotherapy (IMT), and electrodynamic therapy (EDT). Encouragingly, the application of metal catalysts and GOx in synergistic therapy is feasible. Synergistic therapy can leverage the advantages of multiple treatments, resulting in stronger efficacy compared to monotherapy [11]. Thus, synergistic therapy mediated by the GOx-metal catalyst system (G-M) represents a powerful approach against cancer.

Despite its high activity in tumors, GOx suffers from instability, immunogenicity, and a short half-life in vivo [12]. Similarly, addressing the adequate solubility of metal catalysts as tumor therapeutic agents in vivo and activating enzyme activities is crucial [13]. Nanodelivery systems offer the potential to enhance therapeutic efficacy by improving the solubility of therapeutic agents, enhancing in vivo distribution, and enabling targeted delivery [14]. Upon reaching the tumor, these systems can achieve specific activation of G-M activity. Additionally, nanodelivery systems provide a platform for combining G-M with other therapeutic agents to form nanoparticles (NPs). These therapeutic agents primarily include CT drugs, photosensitizers (PTAs), photothermal agents (PSs), sonosensitizers (SSs), immune adjuvants, and more. NPs based on G-M encompass various types, such as metal–organic framework (MOF) NPs, biomineralization-based NPs, metal oxide NPs, noble metal NPs, dendritic mesoporous silica (DMSN) NPs, and polymer NPs. Notably, certain metal catalysts possess both enzyme-like activities and the ability to directly load GOx, facilitating effective integration and controlled release within the same NPs [15, 16]. Therefore, NPs based on G-M hold significant potential as cancer synergistic therapeutic agents.

**Scheme 1.**
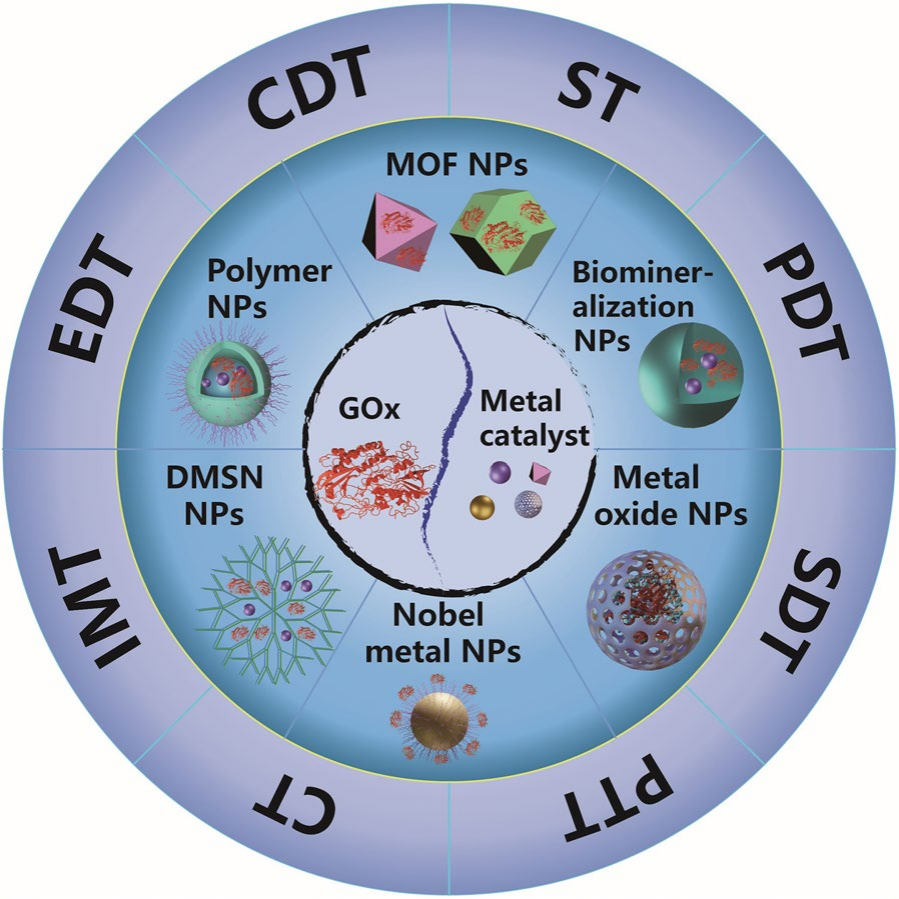
G-M-based nano-delivery systems for synergistic therapy involving ST, CDT, EDT, IMT, CT, PTT, SDT, and PDT. In this synergistic therapy strategy, the constructed NPs include MOF NPs, biomineralization-based NPs, metal oxide NPs, noble metal NPs, DMSN NPs, and polymer NPs. (The permutation in the diagram does not contain an ownership relationship. For example, the two NPs under the ST block do not only complete the ST)

This review provides a summary of the mechanisms by which G-M catalytic combinations achieve synergistic therapy in cancer therapy. It outlines the synergistic therapeutic strategies achievable through various chemical reactions mediated by G-M. Then, it introduces advancements in Fenton reactions and O_2_ enhancement relevant to multimodal synergistic therapy. Furthermore, the review offers a comprehensive discussion of nanodelivery systems based on G-M under different carriers. Ultimately, the nanodelivery system utilizing the catalytic combination of G-M for synergistic therapy represents a promising strategy in cancer treatment today (Scheme 1).

## G-M in tumor synergistic therapy

Synergistic therapy is an excellent strategy that combines the advantages of multiple treatment modes to address tumors through super-additive effects [17]. With multi-pathway synergistic interventions, tumor heterogeneity and complexity are more likely to be overcome.

**Scheme 2.**
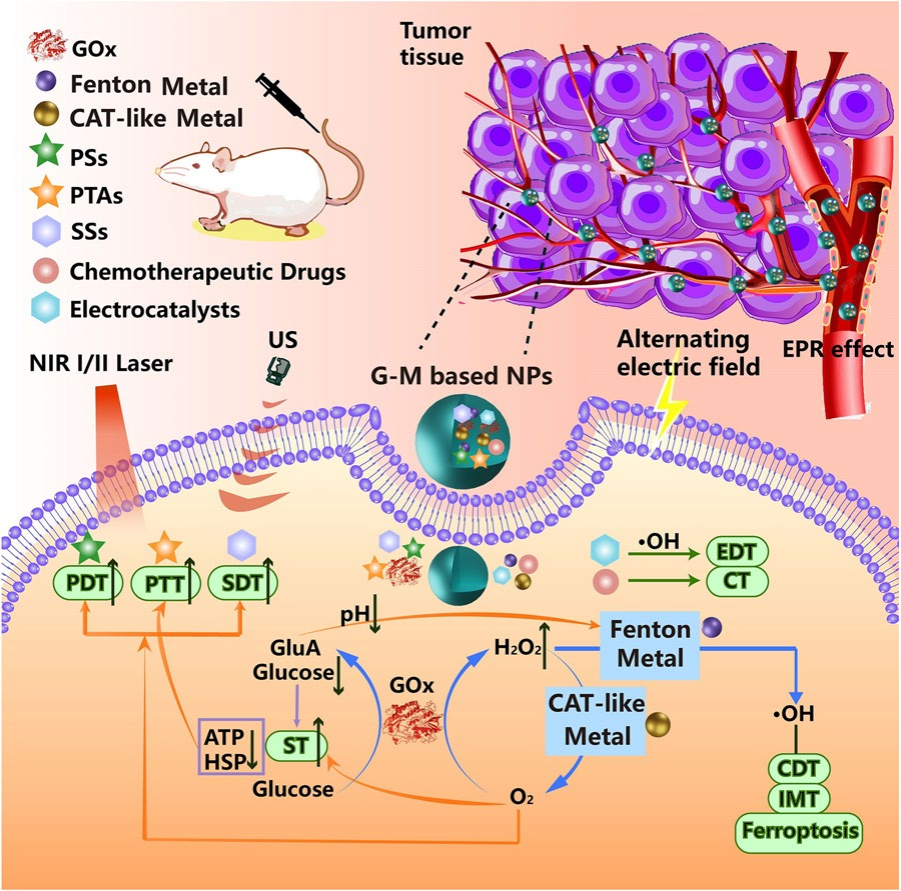
Synergistic therapy mechanism of G-M from different therapeutic agents. G-M NPs enter the tumor and release the therapeutic agents. GOx promotes glucose oxidation to achieve ST, which reduces pH and produces excessive H_2_O_2_. Meanwhile, the effect of PTT is enhanced with a decrease in ATP and heat shock protein (HSP) expression. Due to the catalysis of CAT-like and Fenton metals, H_2_O_2_ acts as a substrate for the next catalytic reaction, contributing to the generation of hydroxyl radicals (**·**OH) and O_2_. O_2_ production can enhance PDT and SDT. Moreover, the generation of **·**OH forms the basis of CDT and IMT and provides the possibility for ferroptosis. Moreover, PTT and PDT are induced by near-infrared light (NIR) I/II. SDT is generated by ultrasound (US). EDT and CT can be combined with the G-M catalytic systems to achieve a more diverse synergistic therapy

Recent developments in catalyst-mediated cancer therapy have opened up new directions for cancer treatment. Catalytic chemical reactions can provide sufficient conditions for synergistic therapy through their rapid catalytic rate. The combination of G-M employs cascade catalysis to ensure multi-pathway synergistic therapy. The nanodelivery system ensures that G-M NPs are selectively enriched at the tumor site and trigger an in situ cascade catalytic reaction [18]. The mechanism and progress of synergistic therapy have been introduced in Scheme 2.

### Mechanism of G-M-induced tumor therapy

The proper integration of the catalytic reaction of G-M is the key pathway for multi-site modulation and tumor removal in synergistic therapy. In a cascade catalytic reaction, multiple treatment modes are rationally combined to produce mutually promoting effects [19]. Metal catalysts interact with GOx primarily through two enzymatic activities: CAT-like activity and Fenton/Fenton-like activity. When the generation of H_2_O_2_ mediated by GOx is utilized by metal catalysts, it leads to the production of O_2_ or** ·**OH, which may result in tumor ablation or enhance other therapeutic effects.

#### GOx-induced glucose consumption and O_2_ production

The development of tumors depends on the energy supply of the glycolysis pathway, making glucose consumption an important target for tumor inhibition [5]. GOx has been extensively used in glucose sensors and nano formulations for diabetes research, owing to its glucose sensitivity and glucose-specific consumption [20, 21]. Recently, the function of GOx for rapid glucose depletion has been recognized in the tumor therapy field, especially for ST [22]. The formula of the GOx-mediated catalytic reaction is as follows [23]:

$$ {\text{Glucose }} + {\text{ O}}_{{\text{2}}}  + {\text{ H}}_{{\text{2}}} {\text{O}}~\xrightarrow{{{\text{GOX}}}}{\text{Gluconic acid }} + {\text{ H}}_{{\text{2}}} {\text{O}}_{{\text{2}}}  $$

In the presence of O_2_, GOx can consume large amounts of glucose, producing gluconic acid and H_2_O_2_. The pH and temperature of cancer cells provide suitable catalytic conditions for GOx. However, hypoxia caused by abnormal tumor growth limits the further catalytic reaction [24].

To address the problem of O_2_ consumption, hemoglobin (Hb), an O_2_ carrier, can deliver O_2_ to replenish the substrate [25]. Nonetheless, the issue of O_2_ availability persists. The role of GOx in cancer therapy is prominent due to the production of H_2_O_2_, which acts not only as a toxic reactive oxygen species (ROS) but also as a substrate that undergoes further catalytic reactions with the assistance of other catalysts. Specifically, metal catalysts with catalase-like activity can continue to release O_2_ by utilizing H_2_O_2_, the product of GOx. The formula for this reaction is as follows [26]:

$$ {\text{H}}_{2} {\text{O}}_{2} + {\text{ CAT}} - {\text{like metal catalysts}} \to {\text{H}}_{2} {\text{O }} + {\text{ O}}_{2} $$

The chemical reaction mentioned produces H_2_O and O_2_ as products, and the generated O_2_ ensures the cyclic operation of ST mediated by GOx. The benefits of CAT-like metal catalysts are as follows [27–29]: (1) Through the cyclic consumption and generation of H_2_O_2_ and O_2_, GOx and CAT-like metal catalysts can achieve efficient self-supplied ST with O_2_. (2) H_2_O_2_ is a product that limits the oxidation reaction of GOx. When the H_2_O_2_ concentration reaches 200 Mm, the activity of GOx is reduced by 40%. (3) Hypoxia has been reported to be a significant factor in cancer invasion and metastasis. Therefore, the method of O_2_ generation provides a reliable means to restore the typical physiological environment of tumor tissue and suppress tumor development. Simultaneously, the process of O_2_ generation can enhance the therapeutic efficacy of other therapies.

Typically, ST is combined with PDT to achieve a synergistic therapy mode of ST/PDT. PDT is a noninvasive therapy that relies on exogenous light stimulation to produce cytotoxic ROS [30]. PSs used in PDT include chlorin e6 (Ce6), methylene blue, and indocyanine green [31]. The mechanism of PDT involves the absorption of photons by PSs, converting them into a transient singlet state. This nonpersistent singlet state is partially transformed into excited triplet states with a longer lifetime through intersystem crossing [32]. The excited triplet states transfer energy and utilize molecular oxygen (^3^O_2_) to produce ROS through two pathways. One pathway involves direct reaction of PSs with organic molecules, leading to the generation of ROS (superoxide anion, **·**OH, or H_2_O_2_) upon contact with O_2_. The other pathway involves direct energy transfer from the excited state PSs to ^3^O_2_, resulting in the highly reactive singlet oxygen (^1^O_2_) [33]. Therefore, adequate O_2_ support is required for the successful progress of PDT. The G-M-mediated O_2_ generation precisely compensates for the O_2_ required for PDT and enables ST/PDT to effectively combat tumors.

Similarly, the combination treatment of SDT/ST can be achieved. SDT, derived from PDT, utilizes exogenous ultrasound (US) at the tumor site to generate ROS through SSs with the presence of O_2_ [34]. The primary types of SSs include porphyrin-based SSs and xanthene-based SSs [35]. Under the US, the SSs release energy during the process of returning to the ground state after being activated, and this energy is transferred to O_2_ to produce ^1^O_2_ [36]. In conclusion, G-M-mediated ST can promote PDT and SDT, and synergistically enhance their therapeutic effects.

#### GOx-enhanced ·OH production

Compared with normal cells, the higher proliferation rate of cancer cells can lead to increased production of ROS [37]. Despite the presence of abundant ROS, cancer cells possess rich reducing substances to regulate intracellular redox homeostasis. Therefore, excessive ROS accumulation can induce oxidative stress in cancer cells, resulting in senescence and various forms of cell death, including apoptosis, necrosis, and ferroptosis [38, 39]. Among different ROS, such as H_2_O_2_, singlet O_2_, and superoxide anion, **·**OH is considered the most toxic ROS with a potent tumor-killing effect [40].

CDT relies on the Fenton or Fenton-like reaction to generate **·**OH within tumors, thereby inhibiting tumor proliferation [41]. Typically, CDT employs nanodelivery systems to transport Fenton agents into tumors, facilitating the conversion of H_2_O_2_ into highly cytotoxic **·**OH. Common Fenton and Fenton-like agents used in CDT include ferrous iron, cuprous ions, manganese ions, copper ions, molybdenum ions, and others [42, 43]. The chemical formula of Fenton and Fenton-like reactions is as follows [26]:

$$ \left( {{\text{M}}^{{\text{n}} + } } \right) \, + {\text{ H}}_{2} {\text{O}}_{2} \to \left( {{\text{M}}^{\left[ {{\text{n}} + {1}} \right] + } } \right) \, + {\text{ OH}}^- + \cdot {\text{OH}} $$

where n is any integer and M represents any metal capable of existing as cations in the n and n + 1 oxidation states. Adequate accumulation of Fenton/Fenton-like agents and H_2_O_2_ can lead to significant **·**OH production. Although the overall reaction does not involve O_2_, a small amount of O_2_ is produced in the intermediate reaction [44].

The accumulation of **·**OH leads to increased lipid peroxidation in cancer cell mitochondria and other subcellular structures, resulting in cytotoxicity [45]. However, the concentration of endogenous H_2_O_2_ (ranging from 10 × 10^−6^ M to 1 × 10^−3^ M) is typically low, resulting in insufficient catalytic kinetics for the Fenton reaction [46]. The optimal pH range for the Fenton reaction is between 2 and 4, while the pH of tumors typically ranges from 5 to 7 [47, 48]. Consequently, efficient **·**OH production at the tumor site necessitates additional H_2_O_2_ and an acidic environment. GOx-guided generation of gluconic acid and H_2_O_2_ effectively addresses these requirements. GOx mediates ST while enhancing metal Fenton agent-mediated CDT, thereby forming a dual-mode synergistic therapy of ST/CDT.

Interestingly, the substantial ·OH production resulting from G-M-mediated ST/CDT treatment is closely associated with tumor immunity, ferroptosis, and cuproptosis.**·**OH can modulate the distribution of tumor-infiltrating immune cells, thereby enhancing immune-mediated tumor clearance [49]. Additionally,**·**OH can induce ferroptosis or cuproptosis in cancer cells with the assistance of related metal catalysts.

IMT relies on the body’s immune function to eliminate tumors. However, the generally low immunogenicity of tumors makes it challenging for the body to mount sufficient immune responses, leading to the failure of tumor immunotherapy, as well as recurrence and metastasis [50]. To address this issue, researchers have discovered that apoptosis serves as a means to activate the immune response [51]. ST/CDT-mediated **·**OH production induces apoptosis, which is a desired outcome of the therapeutic process. During apoptosis, tumor cells undergo immunogenic cell death (ICD) when stimulated by ICD inducers [52]. These inducers encompass chemotherapy drugs, ROS, and oncolytic compounds [53]. Upon stimulation, cancer cells release calreticulin (CRT), ATP, heat shock proteins (HSP), and high mobility group box 1, which facilitate the uptake of tumor antigens by antigen-presenting cells [54]. Consequently, this process triggers cytotoxic T lymphocyte aggregation and immune responses at the tumor site. Ultimately, systemic adaptive antitumor immune responses are activated, establishing long-term immune protective mechanisms that enhance IMT [55]. Therefore, G-M-mediated ST/CDT can synergistically cooperate with IMT to strengthen the treatment effect. Moreover, the accumulation of ·OH in cancer cells leads to ferroptosis, a form of cell death dependent on iron metabolism and lipid ROS.

In conclusion, G-M-mediated synergistic therapy offers a cascade catalysis approach that combines diverse metal catalytic activities to achieve multimodal synergistic therapy. Moreover, G-M has the potential to facilitate even more complex and diverse multimodal synergistic therapies beyond the ones discussed above.

### Advances in the synergistic therapy of G-M

G-M mediated synergistic therapy relies on the Fenton agent/Fenton-like and CAT-like activities of metal catalysts to achieve multipurpose synergistic therapy. Additionally, other catalytic properties of metals, such as electrocatalytic metal-induced EDT, have also been introduced in tumor therapy [56]. Nanodelivery systems combine G-M with various therapeutic agents, including CT drugs and PTAs [57, 58]. The G-M-based synergistic therapy primarily involves Fenton-reaction-related and O_2_-enhanced synergistic therapy. The various treatments and their brief descriptions are summarized in Table 1.

**Table 1:** Differences between the various synergistic therapies

Therapy model	Initiator	Restricted condition in therapy	Tumor-killing pathways	Representative Refs.
ST	GOx	Glucose and O_2_	Consumption of glucose	[59]
CDT	Fenton agent	H_2_O_2_	ROS	[46]
PDT	Light/PSs	O_2_	ROS	[33]
SDT	US/SSs	O_2_	ROS	[37]
EDT	Electricity/electrocatalyst	H_2_O	ROS	[56]
PTT	Light/PTAs	/	Heat	[58, 60]
CT	Chemotherapy drug	/	Direct or simplified inhibition or killing	[57, 61]
IMT	ICD inducers or others	/	Antitumor immunity	[48]

/: none

#### Fenton reaction-involving synergistic therapy

In 1894, French scientist Fenton discovered that Fe^2+^ and H_2_O_2_ could degrade tartaric acid at pH 2.0–4.0, a reaction known as the Fenton reaction [62]. However, the physiological pH value is 7.4, and the concentration of H_2_O_2_ is low [63]. Thus, triggering the Fenton/Fenton-like reaction is challenging. In tumors, the slightly acidic environment and high concentration of H_2_O_2_ make the Fenton reaction more favorable. GOx can generate abundant H_2_O_2_ and increase the acidity of the tumor site, providing essential reaction conditions for the Fenton reaction.

CDT is a non-invasive treatment mediated by the Fenton reaction using H_2_O_2_ as a substrate. The G-M catalytic system supplies H_2_O_2_ generation and facilitates the immediate occurrence of the Fenton reaction. G-M is integrated into NPs to achieve ST/CDT synergistic therapy. In recent years, CT drugs, PSs, and ICD inducers have been co-employed in ST/CDT-based synergistic treatments.

##### ST/CDT/CT synergistic therapy

In the ST/CDT therapeutic system, CT drugs can also be part of the treatment approach. However, the low selectivity of traditional CT drugs often leads to severe adverse reactions in patients [64]. Nanotechnology can improve the aggregation of chemical drugs and combine them with the G-M catalyst system to realize ST/CDT/CT synergistic mode.

Doxorubicin (DOX), a broad-spectrum CT drug commonly used in clinical practice, exerts its effects by inhibiting nucleic acid synthesis [65]. DOX emits red fluorescence upon excitation with 480 nm light, enabling fluorescence imaging. Huang’s group loaded GOx into DOX NPs (PGC-DOX) using CuCaP to achieve ST/CDT/CT therapy [66]. Tumor cells, abundant in glutathione (GSH), convert Cu^2+^ released by the NPs into Cu^+^. As GSH levels deplete, cellular oxidation increases, enhancing CDT. The Cu^+^-guided Fenton reaction exhibits a high reaction rate (1 × 10^4^ M/s) and generates abundant **·**OH [67]. In vivo fluorescence imaging confirmed the significant accumulation of NPs at the tumor site, with the signal remaining high up to 96 h post-injection. PGC-DOX NPs showed significant tumor inhibition compared to Cu^2+^ or DOX alone. Intratumoral and intravenous administration of NPs demonstrated excellent inhibitory effects.

##### ST/CDT/CT/IMT synergistic therapy

In recent years, IMT has shown promising results in cancer therapy by mobilizing the body’s immune cells to activate the immune response against tumor growth and invasion. However, the low immunogenicity of tumors often leads to treatment failure [68].

Li and colleagues modified a MOF with cancer cell membranes overexpressing CRT and combined the delivery of GOx, hemin, and epirubicin (EPI) to create mEHGZ NPs that enhance the therapeutic effect of programmed cell death protein 1 antibody [69]. Apoptosis induced by EPI and CRT on the NP surface promoted the recruitment of immune cells. The iron content in hemin triggered a rapid increase in ROS. Homology recognition of tumor cell membranes facilitated the uptake efficiency of NPs by cancer cells. The data revealed significantly increased ROS levels caused by hemin. In vitro and in vivo experiments confirmed that nanosystems promoted the proliferation of bone marrow-derived dendritic cells and mature macrophages. Combining NPs with programmed cell death protein 1 blockade increased the level of CD8^+^ cytotoxic T lymphocytes from 55.4% to 64.5%, demonstrating the potentiation effect of immunotherapy. The enhanced tumor inhibition rate confirmed the significant antitumor effect of NPs.

##### ST/CDT-mediated targeted ferroptosis or cuproptosis synergistic therapy

Copper (Cu) and iron (Fe) are trace elements in the human body, and their concentrations in cells can cause cell death if the threshold is exceeded [70]. This is also true in cancer cells, where metal ions influence the occurrence and intensity of ferroptosis or cuproptosis [71]. Inspired by this, nanodelivery technology has been used to selectively accumulate metals in tumor tissues, releasing metal ions locally and inducing related forms of cell death [72].

Ferroptosis, as a form of cell death, has emerged as a new therapeutic target for tumors [73]. The accumulation of **·**OH by cancer cells triggers ferroptosis, which depends on iron metabolism and lipid ROS. Once intracellular GSH is depleted, glutathione peroxidase 4 (GPX4) is inactivated, and lipid peroxides cannot be metabolized by GPX4-mediated reactions [74]. Subsequently, ferrous iron further oxidizes lipids through the Fenton reaction, promoting ferroptosis [75]. Iron-based Materials of Institute Lavoisier (MIL) can rapidly release a large amount of ferrous ions after delivery to lysosomes/endosomes in tumor cells [75]. Wang and colleagues loaded GOx onto MIL and modified it with cancer cell membranes to obtain NMIL-100@C NPs with homologous targeting ability [76]. Cell viability assays using several ferroptosis inhibitors (reducing substances) on cancer cells showed significantly higher cell viability compared to the NMIL-100@C group alone. As a result, NMIL-100@C NPs achieved effective ST/CDT synergistic therapy for ferroptosis by inducing the ferroptosis pathway.

Cuproptosis is a unique cell death mechanism that depends on Cu and mitochondria. Excess Cu can bind to lipoylated enzymes in mitochondria, triggering the aggregation of dihydrolipoamide S-acetyltransferase (DLAT) and causing toxicity in cancer cells [77]. Xu and colleagues hypothesized that depleting glucose and GSH could promote cuproptosis and designed ST/CDT/PTD synergistic treatment targeting cuproptosis. They constructed a GSH-responsive GOx-loaded nonporous copper (I) nanoplatform called GOx@[Cu(tz)] [78]. Under NIR-I laser irradiation, this nanoplatform acted as a PTA to generate ROS for PDT. The depletion of GOx and GSH enhanced the binding of Cu (I) to lipoacylase and the aggregation of DLAT. Therefore, GOx@[Cu(tz)] mediated the cuproptosis pathway and achieved a remarkable synergistic effect of PDT/CDT/ST in vitro and in vivo.

##### ST/CDT synergetic PTT or/and PDT

PTT is an exogenous photoinduced therapy that utilizes the photothermal conversion properties of PTAs to ablate tumors [79]. PTT offers non-invasiveness and convenient operation. The NIR-I biological window (750–1000 nm) is commonly used for both PDT and PTT. Recently, NIR-II (1000–1350 nm) has gained attention for its deeper tissue penetration and higher maximum allowable irradiation [80].

Simultaneous use of two types of excitation light enables PTT-enhanced ST and ST-initiated CDT [81]. In this approach, the temperature increase caused by PTT is utilized to promote the catalytic reaction of ST. SrCuSi4O10 NPs with broad NIR absorption are loaded with glucose oxidase (GOx) to achieve ST/CDT/PTT. These NPs, containing the Fenton-like agents Cu and Sr, exhibit absorption properties in both NIR-I and NIR-II regions. The decrease in pH enhances the GOx-mediated catalytic reaction after NIR irradiation. Under the irradiation of both types of light, the NPs demonstrate efficient photothermal conversion.

PDT offers the advantages of minimal trauma, low adverse effects, and precise targeting of tumor tissues [82]. When a material exhibits both PTA and PS properties, PDT and PTT can be combined in a single nanobioreactor. Mesoporous Cu_2_MoS_4_ (CMS), a metal nanostructure, achieves ST/CDT/PTT/PDT synergistic therapy when loaded with GOx to form CMS@GOx [83]. CMS@GOx demonstrates efficient photothermal conversion (63.3%) and superoxide anion generation under NIR-II irradiation. In vivo tumor inhibition experiments show excellent therapeutic efficacy upon light administration. However, an obstacle to the effectiveness of PDT is the availability of O_2_ supply in the tumor. Overcoming the tumor’s hypoxic environment is a key area for further enhancing PDT strategies.

#### O_2_-enhanced synergistic therapy

Hyperbaric O_2_ inhalation was previously considered a solution to alleviate tumor hypoxia [84]. However, incomplete vascular growth in tumor tissue reduces the efficiency of oxygenation, and systemic O_2_ input leads to circulatory toxicity [85]. Metal catalysts, with their suitable stability and high catalytic rates in complex physiological environments, offer a solution [80]. CAT-like metal catalysts can promote the production of O_2_ from H_2_O_2_, including manganese dioxide (MnO_2_), copper oxide, and gold [9, 86, 87]. Increased O_2_ concentration is necessary for ST, PDT, and SDT to achieve effective therapy. A nanodelivery system composed of G-M can accomplish various ST-related synergistic treatments that self-supplement O_2_.

##### ST/PDT and ST/SDT

The rapid growth of tumors leads to an O_2_-deficient (hypoxic) environment due to increased oxygen consumption [88]. Hypoxia is a characteristic feature of solid tumors and contributes to tumor invasion, metastasis, and treatment resistance, particularly in PDT and SDT. Adequate O_2_ supply is crucial for successful PDT and SDT, as they rely on the production of singlet oxygen (^1^O_2_), which induces apoptosis in the mitochondria and nuclei of cells [87]. To address the issue of oxygen deficiency, Yang et al. developed self-enhanced ST/PDT nanoparticles (rMGB NPs) by adsorbing Ce6-grafted Bull Serum Albumin and GOx onto the surface of MnO_2_ [89]. The authors simulated the tumor environment using H_2_O_2_ and glucose and observed significant production of O_2_ and consumption of glucose under these conditions. In O_2_-deficient conditions, the yield of ^1^O_2_ was significantly higher in the presence of GOx than in the no-GOx group. This is because GOx creates an acidic environment that facilitates the decomposition of H_2_O_2_ catalyzed by MnO_2_. Therefore, the therapeutic advantage of oxygen-enhanced ST/PDT is effectively utilized.

Similar to PDT, SDT also relies on O_2_ as a crucial reactant to locally generate ^1^O_2_ upon US stimulation [37]. Platinum (Pt) exhibits lower cytotoxicity and greater stability in physiological environments compared to MnO_2_ [90]. As a SS, PCN 224 was used to construct MOF carriers, and a delivery system consisting of GOx and Pt NPs was incorporated to form PPGE NCs [91]. Dissolved O_2_ analysis after 60 min revealed a higher relative dissolved O_2_ level (33.52 ± 2.86 mg/L), demonstrating the excellent catalytic activity of Pt. In vivo experiments have demonstrated that ST/SDT effectively alleviated tumor hypoxia and improved the antitumor efficacy.

##### O_2_-enhanced ST/EDT

Electrochemical dynamic therapy (EDT) involves the insertion of electrodes directly into tumors to induce significant pH changes in the surrounding tissue, leading to tumor ablation [92]. However, the limited treatment area and complex electrode configurations restrict its application [93]. In contrast, EDT relies on a direct current to induce the accumulation of hydroxyl radicals (·OH) in tumors, causing oxidative stress and tumor damage. Lu et al. employed an EDT/ST coordination strategy to co-deliver GOx and porous Pt nanospheres with electrocatalytic properties to the tumor region [56]. In the presence of abundant chloride ions within tumor cells and the stimulation of an alternating electric field, water (H_2_O) was decomposed into cytotoxic **·**OH. The data demonstrated that **·**OH production was dependent on the presence of Pt, and the rate of glucose consumption was low without Pt. These results indicate that the enhanced GOx reaction under Pt catalysis is attributed to the continuous decomposition of O_2_. Importantly, unlike the previously mentioned therapies (PDT, SDT, CDT), EDT does not rely on H_2_O_2_ or O_2_ to generate ROS. EDT overcomes the limitations associated with exogenous conditions. In conclusion, ST/EDT shows promise as a synergistic therapy for cancer treatment.

##### ST/PTT

PTT relies on NIR irradiation to convert light energy into high heat, effectively eradicating tumors [71]. Melanin, with photothermal conversion and photoacoustic imaging capabilities, significantly enhances the biocompatibility of MnO_2_ and provides GOx ligation sites [94].

Huang's group proposed a novel approach by using MnO_2_ grown with melanin as a template and armed with GOx (MNS-GOx) to achieve O_2_ self-supplied ST/PTT and magnetic resonance imaging (MRI)/photoacoustic imaging [95]. This system completes a cyclic cascade catalytic reaction between ST consumed by glucose and O_2_ generation. Covalently crosslinking MnO_2_ increased the oxidation rate of GOx from 2.69 to 3.43 mM/min. Additionally, the thermal enhancement effect caused by the 808 nm laser improved the efficiency of GOx catalysis. In vivo dual-mode imaging confirmed that MNS-GOx exhibited significantly enhanced MRI contrast effects relative to MNS. The acidity enhancement induced by glucose consumption directly caused the degradation of MnO_2_ and the release of manganese ions, promoting an enhancement of the MRI imaging effect. Therefore, in well-designed tumor treatments, the process of O_2_ release also aids in enhancing imaging.

Tumor heat tolerance plays a pivotal role in the effectiveness of PTT. It has been confirmed that glucose depletion due to GOx hinders ATP production, resulting in a decrease in HSP expression [96]. HSP is a crucial heat-resistant protein derived from heat-stimulated tumor cells that protect themselves from heat damage [97]. Researchers developed liquid metal-based NPs, a type of PTA, loaded with Gox, to achieve ST/PTT with a high tumor suppression rate [98]. Similarly, researchers carefully designed GOx-loaded iron oxide NPs to enhance the effectiveness of magnetic hyperthermia therapy, which is hindered by tumor heat tolerance [99]. The inclusion of GOx and CAT-like metal catalysts in the PTT process shows promise in overcoming heat resistance problems.

The G-M system offers unique advantages in the synergistic therapy of cancer. (1) Synergistic therapy addresses the limitations of monotherapy, solving multiple treatment process problems simultaneously. The nanodelivery system provides G-M to integrate multiple therapy modalities into one system for more efficient treatment. (2) G-M-mediated catalytic reactions provide multifarious treatment strategies for achieving multi-functionalization, such as self-enhanced ST, PDT, and SDT. Additionally, Fenton agent-based G-M can use ROS to target specific pathways of cancer cells, leading to ferroptosis, cuproptosis, or ICD. Particularly, the G-M-induced ICD effect is crucial for enhancing IMT. (3) G-M can directly initiate a cascade catalytic reaction, fully utilizing the product H_2_O_2_ to achieve the purpose of O_2_ supplementation and ROS increase. (4) Notably, G-M exhibits advantages in tumor imaging, such as MRI. Ultimately, the inclusion of nanodelivery technology further enhances the effectiveness of G-M-mediated synergistic therapies.

## NPs for delivering G-M

The occurrence and progression of tumors involve complex regulatory mechanisms and multiple mechanisms to evade apoptosis [100]. Synergistic therapies based on multiple therapeutic agents are available to address tumor complexity. However, delivering multiple therapeutic agents simultaneously has the disadvantage of inconsistent pharmacokinetics [101]. Nanodelivery technology offers a solution by co-loading various therapeutic agents in the same nanosystems, achieving simultaneous delivery in the same time and space [102].

Nanocarriers improve the stability of delivered therapeutic agents and provide targeting and controlled release functions [103]. Generally, these NPs accumulate at the tumor site through enhanced permeability and retention effects, enabling the responsive release of therapeutic agents [104]. Passive targeting relies on the size effect of NPs, where NPs smaller than 200 nm can avoid phagocytosis by the reticuloendothelial system. Using nanocarriers, G-M can be bound to NPs to achieve diverse tumor synergistic therapeutic purposes. Recently, MOF, biomimetic mineralization, metal oxides, noble metals, DMSN, and polymers have been primarily used as carriers in G-M delivery systems. The design and improvement methods of NPs in G-M combination applications are summarized below, along with an evaluation of the impact and potential problems of vector design strategies on delivery behavior and treatment. In addition, we compared the properties of G-M NPs related nanomaterials in Table 2.

**Table 2:** Performance comparison between different nanomaterials

Nanomaterials	Biocompatibility	Safety	Catalytic performance	Stability	References
MOF	√	√	√	√	[69, 78]
Biomineralization	√	√	—	√	[105]
Metal oxides	√	—	√	√	[95]
Noble metal	√	√	√	√	[58]
DMSN	√	√	—	√	[106]
Polymer	√	√	√	√	[107]

“√” is represented that the reference has been investigated and good results have been obtained

“—” indicates not examined in detail in the reference

### MOF-based NPs

MOF is a nano-sized backbone formed by the coordination of metal and organic ligands and serves as a versatile enzyme nanocarrier with a high loading rate [108]. To construct the GOx and metal catalyst nanodelivery system, MOFs can be self-supplied or doped/loaded with metal catalysts to deliver GOx. The primary types of MOFs used are MIL and zeolitic imidazolate framework (ZIF).

#### MIL-based NPs

MIL is a series of framework structures synthesized using metal ions with different valences and carboxylic acid ligands [109]. In G-M-induced therapy, MIL-based NPs primarily employ MIL-100 to deliver GOx. MIL-100 is a readily modifiable MOF with 1,3,5-benzenetricarboxylic acid as the ligand and Fe^3+^ as the metal node [110]. MIL-100 integrates nanocarriers with Fenton therapeutic function, reducing the unpredictable consequences of involving other nontherapeutic molecules.

Zhang et al. designed MIL-100-based NPs loaded with GOx and modified with hyaluronic acid-polydopamine for active targeted synergistic therapy involving PTT/ST/CDT [111]. Polydopamine was used as a surface-modified PTT therapeutic material due to its good biocompatibility and photothermal conversion properties [112]. Hyaluronic acid targets the highly expressed CD44 receptor in tumor cells, aiding NPs in localizing to tumor regions [113]. In experiments, increased **·**OH concentration and significantly decreased glucose concentration confirmed the efficient ST/CDT achieved by iron ions working with GOx.

It is worth noting that CD44 receptors are also expressed in normal cells, and although CD44 has a poor affinity with healthy cells, it still has the potential to be toxic to normal tissue [114]. The homologous targeting ability of cell membranes has been used for biomimetic modification of NPs in cancer treatment [115]. Wan and colleagues designed MIL-100-based NPs with a high GOx loading capacity (36 g/mg) and camouflaged them with cancer cell membranes, forming NMIL-100@GOx@C [116]. SDS-PAGE demonstrated the successful synthesis of GOx with the cell membranes. In the presence of the NPs, pH rapidly decreased due to the glucose oxidation process. The cancer cell membranes wrapped around MIL-100 enable immune escape and homologous targeting of NPs. In vivo experiments revealed good NP enrichment and antitumor effects. Therefore, MIL-100 achieved satisfactory therapeutic effects with the assistance of active targeting.

#### ZIF-based NPs

The high levels of lactic acid produced by tumor glycolysis contribute to the acidic conditions in tumors, endosomes, and lysosomes [77]. ZIFs are typically prepared using zinc or cobalt as metal nodes and imidazole as ligands [117]. In acidic tumor conditions, the imidazole groups of ZIFs undergo protonation, leading to the dissociation of the coordination bond between the metal and the ligand, causing the collapse of the entire structure. However, loading GOx into ZIFs has been shown to result in early leakage and systemic toxicity [118].

**Fig. 1:**
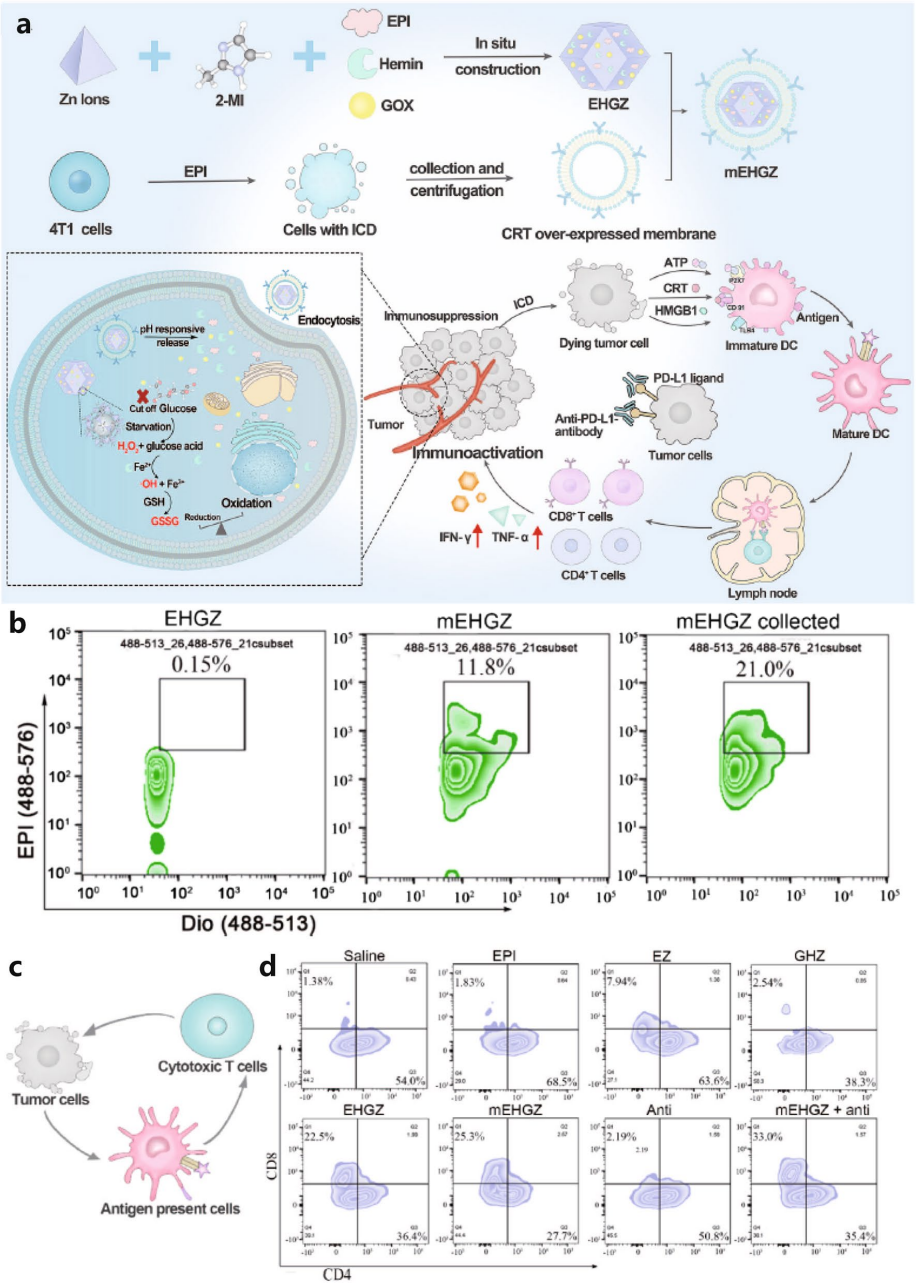
**a** The scheme of the mEHGZ NPs induced IMT-relevant cancer synergistic therapy. **b** Flow cytometry results of the membrane encapsulation efficiency on mEHGZ NPs. **c** Scheme of ICD triggering the release of antigens and DAMPs. **d** Percentage of CD8 + T cells in tumor tissue after different treatments [69], copyright © Bioactive material 2022

Encapsulation with cancer cell membranes can prevent NP leakage and immune system phagocytosis while providing active targeting ability [119]. Importantly, specific protein expression on cell membranes directly affects the functionality of NPs. During early tumor ICD, CRT is rapidly expressed on cancer cell membranes (Fig. 1c) [120]. These cancer cells emit an “eat me” signal, stimulating the immune system to activate and destroy the cancer cells. Hemin, an iron-rich component of human blood and an important Fenton agent [121], was used by Li et al. to encapsulate ICD inducers EPI, GOx, and hemin within ZIF-8, along with cancer cell membrane camouflage that overexpressed CRT, forming mEHGZ NPs (Fig. 1a) [69]. These NPs enabled ST/CDT/IMT synergistic treatment. The NPs had a size of 133.92 ± 2.93 nm. Compared to normal cells, mEHGZ NPs were more than 4 times more likely to be taken up by cancer cells. In vitro cell co-culture experiments revealed significant immune activation effects. As shown in Fig. 1b, the membrane encapsulation efficiency was 21.0%, which increased after centrifugation. The EPI treatment group showed an increase in CD8^+^ T cells from 1.83% to 33.0% in the mEHGZ + anti-PD-L1 antibody treatment group (Fig. 1d). Thus, the modified NPs exhibited significantly enhanced therapeutic effects.

Positively charged NPs interact with negatively charged cell membranes through electrostatic attraction, facilitating higher drug uptake rates and enhancing tumor inhibition effects [122]. Bull Serum Albumin-grafted 4,40-Azonzenecarboxylic acid-modified ZIF-8 NPs were prepared through electrostatic interaction. These NPs loaded with GOx and iron NPs achieved targeted ferroptosis for ST. Under tumor hypoxic conditions, 4,40-azonzenecarboxylic acid spontaneously becomes positively charged, promoting the uptake of NPs by cancer cells [123]. However, compared to covalent modification, the weaker electrostatic forces are more prone to premature shedding in the complex physiological environment [124]. The lack of active chemical groups in the structure of ZIFs is the main limitation affecting the surface functionalization of ZIF NPs. ZIFs cannot guarantee stable functional modification through strong covalent bonding. Therefore, future research should focus on the development of more robust functional modification methods that preserve the activity of GOx and other therapeutic agents.

#### Others

In addition to MOF and biomineralization-based NPs, there are other types of nanoparticles used for the delivery of G-M. Nonporous [Cu(tz)] NPs have been employed to prevent the leakage of GOx and the penetration of small molecules in normal physiological conditions [78]. The compact nanostructure of [Cu(tz)] consists of a tricoordinated triazolic acid ligand and Cu(I), with negligible distances between layers. Xu and colleagues encapsulated GOx in [Cu(tz)] to form GOx@[Cu(tz)] NPs for synergistic ST/CDT therapy with cuproptosis [84]. This structure prevented enzyme inactivation and systemic toxicity caused by the infiltration of blood glucose and O_2_. Various experiments demonstrated significant glucose oxidation and Fenton catalytic performance in simulated tumor conditions while showing minimal response under physiological conditions.

GOx, being a protein-based enzyme, is susceptible to external physical or chemical factors that can affect its folded structure [125]. Nonporous MOFs, such as [Cu(tz)], provide a new approach to preserving enzyme activity. Additionally, the use of water as a solvent for MOF preparation avoids the impact of organic solvents on the GOx structure. However, as a metal material, MOFs need further improvement in terms of biocompatibility. While many MOF-based delivery systems have been developed, the construction of versatile and biocompatible MOFs remains an ongoing challenge. Such carrier materials hold the potential to offer new possibilities for G-M synergistic therapy, enhancing the effectiveness of nanotherapeutics in cancer treatment and reducing side effects.

### Biomineralization-based NPs

Biomineralization is a biochemical process that occurs widely in nature, involving the nucleation and growth of inorganic matter based on organic templates [126]. For instance, human bones grow calcium phosphate (CaP) using collagen fibers as templates [127]. During mineralization, other metal ions or organics can be incorporated for the co-delivery of various therapeutic agents. Biomineralization-based nanoparticles possess good biocompatibility and controllable morphological characteristics [128]. The main forms of biomineralization-based nanoparticles include CaP and calcium carbonate (CaCO_3_).

#### CaP-based NPs

CaP is a common component of hard biological tissues in the human body, known for its excellent biocompatibility and biodegradability [129]. Importantly, CaP can degrade into nontoxic phosphate and Ca in the acidic tumor microenvironment and participate in normal metabolism. To prepare NPs, GOx can be used as a template and incubated with Ca^2+^ and PO_4_^3+^ ions in a cell culture medium for 24 h at 37 °C [130]. Under these conditions, the negatively charged GOx induces the aggregation of surrounding Ca^2+^ ions, resulting in the formation of biomimetic mineralized NPs.

Huang’s group developed Cu-doped CaP mineralized polyethylene glycol (PEG)-modified GOx (PGC) and loaded it with DOX to obtain monodisperse PGC-DOX NPs [66]. These nanoparticles enabled multimodal synergistic treatment involving ST/CDT/CT. The researchers found that the modified GOx in PGC showed better dispersion homogeneity compared to unmodified GOx. Circular dichroism spectra demonstrated that the modified GOx did not undergo structural changes. The catalytic activity of PGC reached equilibrium within 1 h under different glucose concentrations, as indicated by the concentration of H_2_O_2_ produced. The average particle size of PGC-DOX NPs was 88 ± 17 nm. The concentration dependence of ·OH on glucose was observed through the 3,3′,5,5′-tetramethylbenzidine assay, revealing its concentration-dependent absorbance value. Hemolysis experiments demonstrated good biocompatibility and safety, with a hemolysis rate of less than 3.0%. In vitro fluorescence imaging revealed the fluorescence signal of PGC-DOX nanoparticles in tumors for up to 144 h, indicating prolonged in vivo action.

#### CaCO_3_-based NPs

CaCO_3_ is another commonly used biocompatible material in nanodelivery technology [131]. The mineralization of Ca and GOx can be utilized to prepare CaCO_3_ shells, which protect the activity of enzymes not fixed inside NPs.

Tannin, with its molecular structure containing numerous hydroxyl groups, can form multinucleated coordination complexes with polyvalent metals, providing opportunities for protein loading [132]. However, proteins often suffer from encapsulation through weak interactions, leading to leakage issues. Biomimetic mineralization addresses this problem. Yin and colleagues employed the polyhydroxyl properties of tannin to crosslink Mn^2+^ and immobilize protein drugs as well as GOx, which were subsequently protected with CaCO_3_ [133]. Interestingly, the exposed hydroxyl groups after tannin crosslinking facilitated the aggregation of calcium ions, accelerating biomimetic mineralization and reducing particle size. The particle size of the protein-loaded Mn-tannin NPs significantly decreased from 459 ± 42 nm to 165 ± 51 nm after mineralization. The encapsulation rate of GOx was 13.1 wt%. Unlike the 7.6% release rate at pH 7.4, it exhibited a release characteristic of 31.6% at pH 5.0. The release of **·**OH from the NPs was dependent on H_2_O_2_ concentration, reaching up to 93.916%.

In addition to the above, biomineralization-based nanoparticles can take on other forms. Through biomineralization, GOx can also be combined with zero-valent iron or Mn^2+^ to achieve ST/CDT or IMT/ST synergistic modes [134]. These formulations also demonstrated promising outcomes.

Overall, biomineralization-based NPs utilize metal ions to construct GOx-loaded nanocarriers, offering the advantages of simplicity, mildness, and safety. These NPs can accumulate at the tumor site to exert therapeutic effects and degrade into low-toxicity substances in the body. Moreover, biomimetic mineralized nanoparticles can introduce other functional metal ions to achieve efficient cascade catalytic therapy in tumors. Biomineralization-based NPs have emerged as promising candidates for future G-M catalytic systems in synergistic cancer therapy.

### Metal oxides-based NPs

Metal oxides are commonly employed therapeutic catalysts in nanodelivery systems for synergistic therapy. Interestingly, some metal catalysts are also materials used for immobilizing natural enzymes, such as iron oxide and CuO [18, 102]. They can be fabricated into nanodelivery systems with diverse nanostructures, catalytic properties, and biological functions. Metal oxides can provide Fenton and CAT-like activities, thereby facilitating ST/CDT or enhanced-ST processes. They can combine with GOx through hole embedding, encapsulation, and conjugation to deliver therapeutic molecules to the tumor site.

Hollow structures with internal cavities can encapsulate more GOx, leading to enhanced catalytic effects. Ying et al. designed hollow iron oxide nanocatalysts (HIONCs) for the effective immobilization of GOx and accomplished ST/CDT [135]. They first etched iron oxide with hydrochloric acid for different durations and determined the change in iron content and structural properties. In HIONCs-GOx, GOx achieved an encapsulation rate of 60% and a loading rate of 16.6%. The high loading capacity may be attributed to GOx being loaded onto HIONCs through covalent binding, surface adsorption, and encapsulation. Despite the loss of some iron after etching, the production rate of ROS and O_2_ significantly increased.

Indeed, the biocompatibility of metal carriers is generally limited, but it can be improved through chemical modifications to enhance their in vivo compatibility. Wang et al. developed hollow mesoporous cupric oxide loaded with GOx and coated with polydopamine to create HMCGP NPs for ST/CDT [100]. GOx achieved a loading capacity of up to 47.1%. The polyhydroxy structure of polydopamine provides chelating sites for metal NPs, thereby improving their compatibility. Polydopamine also exhibits good hydrophilicity and pH degradation properties, which enhance the dispersion and selectivity of the modified NPs. Cytotoxicity and hemolysis tests confirmed the favorable biocompatibility of the NP carriers. Cupric oxide displayed CAT-like activity, and it degraded into monovalent copper ions, serving as a Fenton agent. In the simulated physiological environment of the tumor, the rate of ROS production was significantly increased. Chemical modification effectively addressed the issues of poor biocompatibility and stability associated with metal oxide-based NPs.

**Fig. 2:**
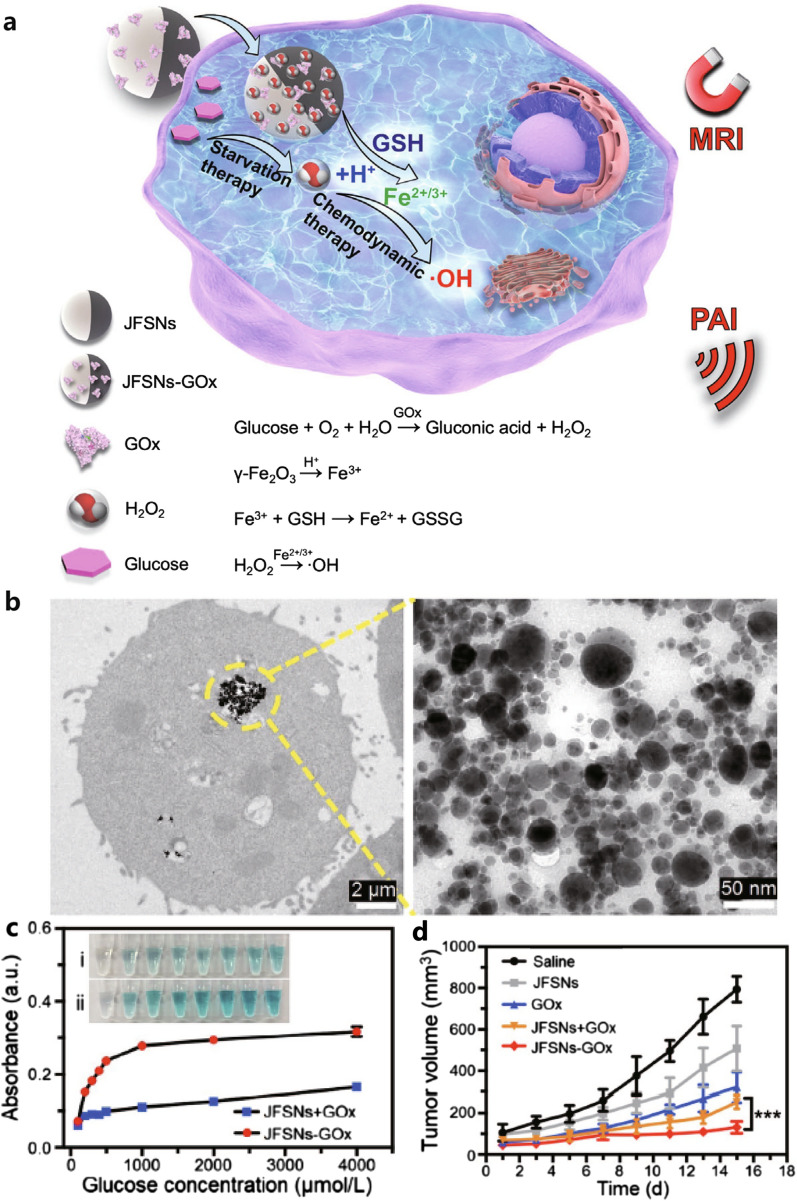
**a** Scheme of the JFSNs-Gox-induced ST/CDT cancer synergistic therapy. **b** The TEM images of cancer cells incubated with the NPs for 1 day (left panel) and NPs morphology (right panel). **c** Fenton catalytic property. **d** Tumor growth curves of 4T1 tumor-bearing mice under different treatments [135], copyright © Science Bulletin 2022

In nature, the “substrate channel effect” refers to the phenomenon of multi-enzyme cascade reactions occurring only when two enzymes are in close proximity. However, manufacturing controlled multi-enzyme cascade nanoreactors artificially remains a challenge. Janus particles, which are anisotropic NPs formed by two substances with dual properties aggregated onto a single NP system, can overcome this challenge [136]. Zhang et al. utilized the silicon fraction of Janus-type γ-Fe_2_O_3_/SiO_2_ NPs to conjugate GOx (JFSNs-GOx) and achieved highly effective ST/CDT synergistic therapy (Fig. 2a) [137]. In acid- and GSH-rich tumor cells, γ-Fe_2_O_3_ was initially degraded into ferric iron. Subsequently, GSH reduced trivalent iron to the divalent state, supplying the Fenton agent. Compared to traditional core–shell structures, the distance between GOx and γ-Fe_2_O_3_ in JFSNs-GOx was more appropriate, allowing γ-Fe_2_O_3_ to fully contact the H_2_O_2_ substrate. After incubation with JFSNs-GOx, cancer cells could effectively internalize the nanoparticles while maintaining their shape (Fig. 2b). The rate of **·**OH generation was nearly twofold higher after conjugating GOx to JFSNs compared to free GOx (Fig. 2c). JFSNs-GOx significantly reduced tumor volume compared to the saline group (Fig. 2d).

Moreover, MnO_2_ and titanium dioxide have also been utilized as carriers to co-construct NPs with GOx for synergistic cancer therapy [101]. The structural modification of metal oxides can enhance the loading capacity and cascade catalytic efficiency of GOx, leading to strong development potential. With the assistance of metal oxide supports, the G-M system has achieved satisfactory catalytic and therapeutic outcomes.

### Noble metal-based NPs

Noble metal-based NPs possess unique surface properties and excellent optical and thermal characteristics [138, 139]. These NPs play a significant role in cancer treatment by integrating photothermal conversion performance and catalytic activity. Some gold-based nanomedicines have even received approval from the Food and Drug Administration (FDA) for clinical trials [140]. The CAT-like activity and biocompatibility of noble metals make them crucial metal catalysts for GOx binding [103].

**Fig. 3:**
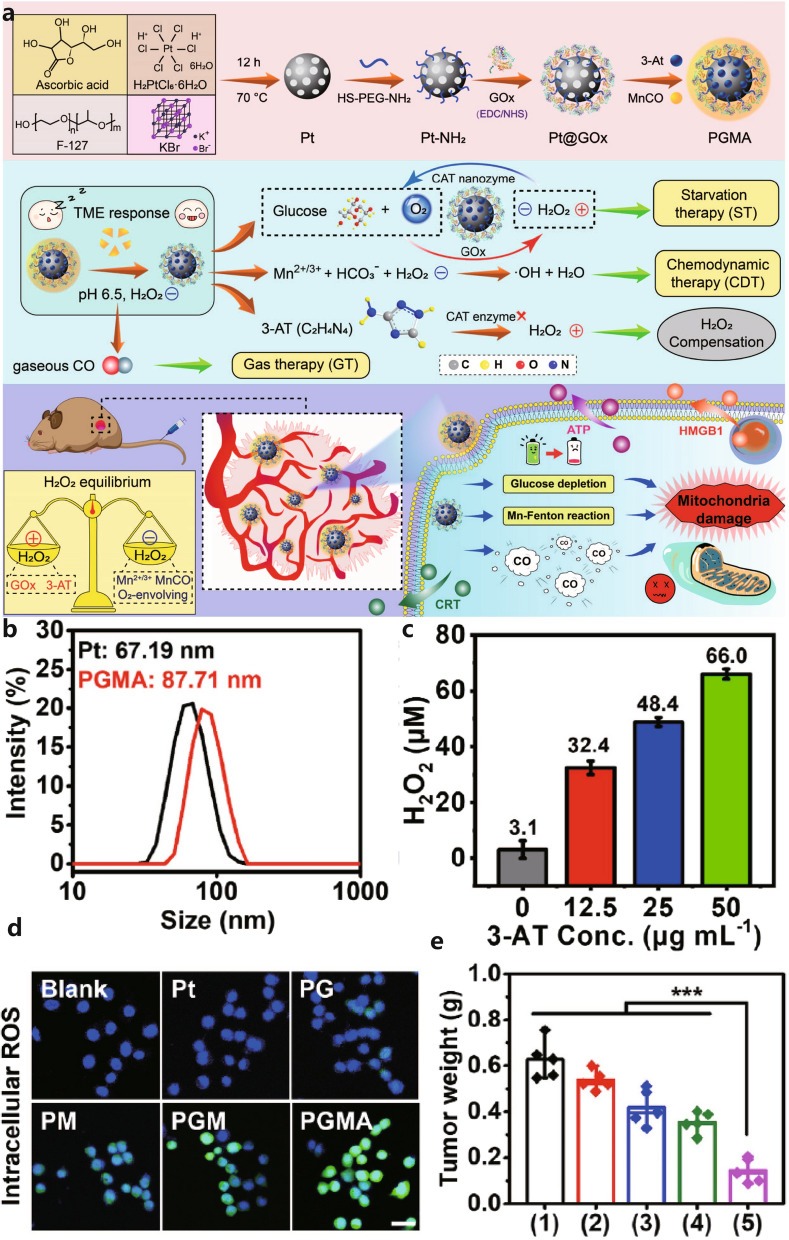
**a** Scheme of the PGMA NPs-induced ST/CDT/GT cancer synergistic therapy. **b** Particle size distribution diagram of Pt NPs and PGMA NPs. **c** H_2_O_2_ concentration changes. **d** Intracellular ROS content. **e**Tumor volume change. [143], copyright © Biomaterials 2022

Gold, silver, and platinum (AuAgPt) NPs are commonly used in PTT due to their excellent photothermal conversion properties [141]. Wang et al. utilized HS-PEG-NH_2_ to modify AuAgPt NPs prepared using the one-pot method and conjugated GOx to achieve O_2_-enhanced PTT/ST/IMT [142]. The enzymatic activity of Pt facilitated the rapid decomposition of H_2_O_2_. Huang et al. used the sulfhydryl chelation of HS-PEG-NH_2_ to chelate mesoporous Pt, with the amino group on the other end covalently binding to GOx via an amide reaction (Fig. 3a). They then wrapped manganese carbonyl (MnCO) and 3-amino-1,2,4-triazole (3-AT) to form PGMA NPs [143]. PGMA NPs enabled synergistic ST/CDT/GT therapy. Under acidic conditions, MnCO was decomposed to CO to kill cancer cells. Mn^2+^ acted as Fenton-like agents, converting H_2_O_2_ to **·**OH. Importantly, Pt competed for the use of H_2_O_2_, releasing sufficient O_2_ to supply GOx for cyclic catalytic reactions. 3-AT inhibited endogenous catalase, thereby enhancing the progression of cascades. The hydrodynamic diameter of PGMA nanoparticles was 87.71 nm (Fig. 3b). The content of MnCO and 3-AT was 15.7% and 8.67%, respectively. According to standard BCA protein assays, the loading efficiency of GOx in PGMA NPs was 2.7%. The concentration of H_2_O_2_ was dependent on 3-AT (Fig. 3c). The experimental results demonstrated the efficient catalytic and therapeutic effects of GOx, Mn^2+^, and Pt (Fig. 3c, d).

Noble metals possess multifunctional properties, acting as PTAs and CAT-like enzymes while serving as carriers for GOx in NP form. Compared to the encapsulation of GOx within metal oxide pores, noble metals can achieve lower loading efficiency through chemical conjugation. Although the content of GOx may be small, the high CAT-like activity of noble metals can increase the oxygen concentration in the GOx-catalyzed reaction and promote the ST process. Ultimately, noble metals with diverse enzyme-like activities and photothermal conversion functions are important candidates for synergistic cancer therapy related to PTT.

### Silicon- and polymer-based NPs

Silicon- and polymer-based NPs have gained attention in the field of nanomedicine due to their diverse properties and high biocompatibility requirements for nanodelivery systems. Silicon, which has an FDA-approved safety profile, shows promise as a nanocarrier [144]. Polymers, on the other hand, are easily modifiable and can combine various functions such as ligand modification for active targeting and responsive functional bond linking [143]. Both silicon and polymers have excellent biocompatibility, making them well-established components of nanodelivery systems. In the context of cancer synergistic therapy, metal catalysts are often utilized in the form of NPs or chelated organic molecules to construct nanodelivery systems in combination with GOx. Mesoporous silica, such as DMSN, can be tailored into nanocarriers with different pore sizes, allowing for efficient encapsulation and delivery of GOx and metal catalysts.

#### DMSN-based NPs

Mesoporous silica has a relatively low loading capacity for macromolecules, and GOx is primarily loaded through chemical conjugation [145]. However, the modification of GOx structure through chemical reactions and the shielding of catalytic sites after modification pose challenges to its further development [146]. DMSN, with its large pore size, can easily accommodate large proteins during preparation, enabling increased loading of GOx [147]. Under normal physiological conditions, DMSN ensures that GOx and metal catalysts maintain excellent catalytic performance while facilitating the proper diffusion of small molecule products and substrates.

**Fig. 4:**
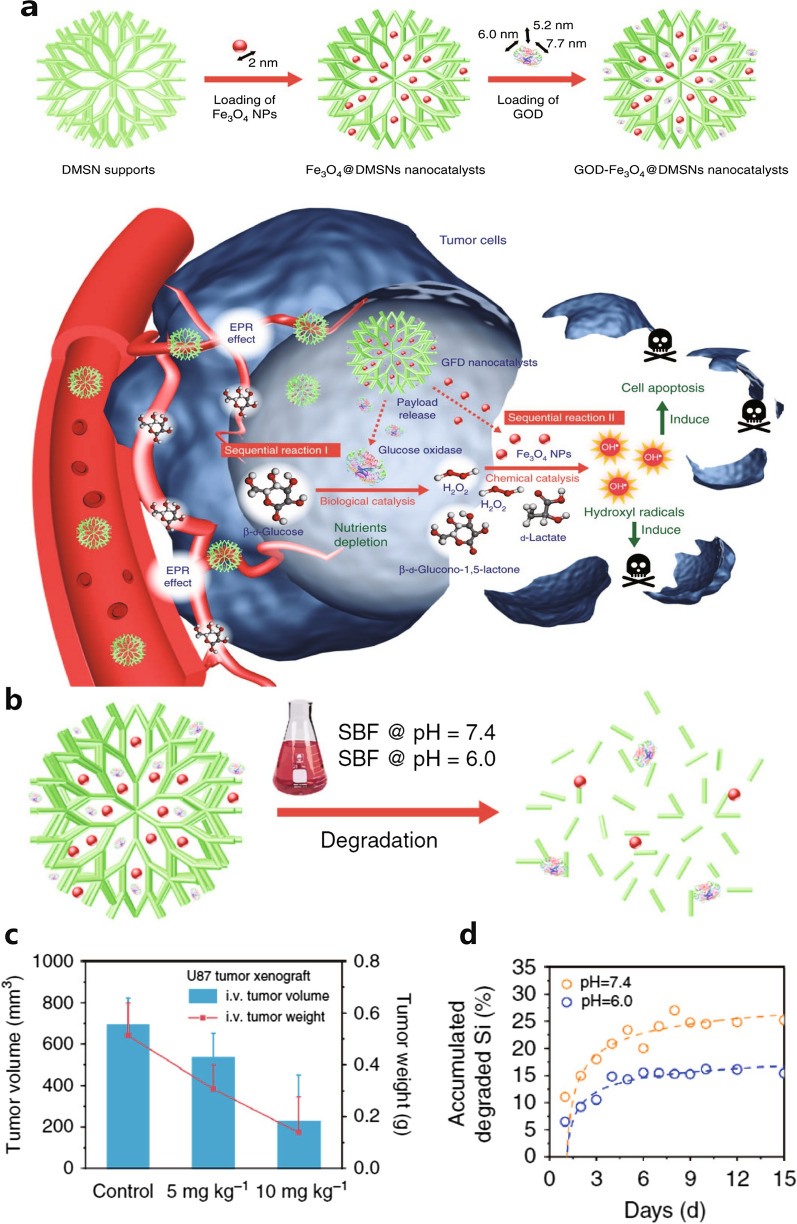
**a** The scheme of the GOD-Fe_3_O_4_@DMSNs-induced ST/CDT. **b** and **d** Biodegradation principle and performance of GOD-Fe_3_O_4_@DMSNs. **c** Changes in the tumor volume and weight under different therapy agent concentrations [106], copyright © Nature 2017

GOx is a biological macromolecule with a hydrated particle size of approximately 7.6 nm [148]. Shi Jianlin’s group constructed a tumor-selective catalytic nanodrug by using large pore size and biodegradable DMSN to load GOx (referred to as GOD) and ultra-small Fe_3_O_4_ NPs (GOD-Fe_3_O_4_@DMSNs) (Fig. 4a) [106]. With a pore size of 40 nm, DMSN enabled co-packaging of GOx and Fe_3_O_4_ NPs with a maximum diameter of 9.7 nm. The loading capacity was remarkable, with GOx and Fe_3_O_4_ reaching 16.61% and 15.87%, respectively. The dual enzyme activities of Fe_3_O_4_ NPs served as Fenton agents and O_2_ producers, enhancing the production of **·**OH. The biodegradability of DMSN was demonstrated in acidic (pH 6.0) simulated body fluid, showing significantly lower degradation rates compared to neutral environments (pH 7.4) (Fig. 4b and 4d). This indicated that the NPs could persist in the tumor for a relatively long time, while rapidly degrading and being excreted in normal tissues. The GOD-Fe_3_O_4_@DMSNs exhibited a significant tumor inhibition effect (Fig. 4c).

Considering the large pore size and spatial structure of DMSN, particle size modification is an important factor to consider in NP design. NPs with large particle sizes are more prone to phagocytosis by the reticuloendothelial system, limiting their effectiveness [149].

#### Polymer-based NPs

Under appropriate molecular structure design, polymers can form NPs through intermolecular forces in aqueous solutions [150]. Amphiphilic polymers, which consist of two or more distinct polymer chains, are easily modified and functionalized [151]. Depending on the assembly methods, polymers can spontaneously form micelles or polymersomes in water [152]. The structure of polymers is highly modifiable, allowing for the chemical bonding of functional groups in response to stimuli and enabling the loading of hydrophilic and hydrophobic therapeutic agents.

Li et al. developed a nanocomposite using POEGMA-b-PTKDOPA as the polymer segment to encapsulate a protein/metal complex for synergistic therapy combining ST and CDT [107]. The polyhydroxyl group of oligomerized ( −)-epigallocatechin-3-O-gallate in this polymer complexed with the protein to immobilize programmed death ligand 1 antibody/GOx. Iron ions were co-chelated with the hydroxyl group of oligomerized ( −)-epigallocatechin-3-O-gallate and the block polymer hydroxyl. The PTKDOPA segment of the polymer was responsive to ROS, causing a rapid release of proteins when intracellular ROS levels increased. The particle size of the nanocomposite was 110.3 ± 7.2 nm, and the protein loading efficiency reached 60% due to the proper control of the hydroxyl content. Polymersomes, formed by amphiphilic polymers, can encapsulate water-soluble drugs in their internal cavities. Wang and colleagues prepared pH-responsive PEG-b-P(FcMAco-PEMA) polymersomes that loaded GOx in the hydrophilic core and the chemotherapeutic drug tirapazamine (TPZ), forming a nanoreactor called GOD/TPZ@PFc for ST/CDT/CT [153]. The loading capacity of GOx and TPZ in the nanoreactor was determined to be 6.4% and 5.2%, respectively, using fluorescence spectroscopy. The particle sizes of the polymersomes at pH 7.4 and 6.5 were measured to be 71 ± 18 nm and 79 ± 11 nm, respectively. Within 24 h, the release rate of TPZ reached 80% due to the acidity enhancement. Hemolytic tests demonstrated the biocompatibility of the polymersomes.

As mentioned earlier, the loading efficiency of GOx varies significantly depending on the design of different nanodelivery systems and the utilization of molecular forces. Even when GOx loading is achieved, the encapsulation rate of the metal catalyst is limited compared to NPs where the metal catalyst serves as the carrier. Therefore, it is necessary to strike a balance between G-M catalytic activity and carrier properties to effectively improve the therapeutic outcomes.

## Conclusions and challenges

In conclusion, this review has summarized the diverse strategies and nanodelivery system designs enabled by G-M synergistic therapy. These NPs can efficiently block glucose uptake while utilizing the oxidation product H_2_O_2_ to trigger cascade reactions with the assistance of metal catalysts. By increasing the concentration of O_2_ or **·**OH, the G-M strategy regulates the tumor hypoxic environment and inhibits cancer cell proliferation. Various nanocarriers have been developed for the delivery of GOx and metal catalysts, including MOF, biomineralization-based NPs, metal oxides, noble metals, polymers, and silicon-based NPs. While G-M synergistic therapy holds promise as an emerging treatment strategy, it also presents challenges that need to be addressed.

### The advantages and disadvantages of G-M mediated synergistic therapy

G-M mediated synergistic therapy offers several advantages compared to traditional treatment modalities such as surgery, radiotherapy and chemotherapy. Some advantages include: Tissue specificity and killing efficiency: Nanocatalytic therapy has high specificity for tumor tissues and demonstrates efficient killing of cancer cells. The rapid catalytic reactions within the tumor tissue leading to substrate consumption and product formation, resulting in physiological and pathological effects specifically within the tumor. Multimodal synergistic therapy: G-M mediated therapy can combine multiple treatment modes such as ST, CDT, EDT, IMT, PTT, PDT, and SDT. This allows for multi-site regulation and killing, enhancing the therapeutic efficacy by targeting tumors through various pathways. Independence from tumor pH: The Fenton reaction, facilitated by GOx and metal catalysts, can proceed optimally regardless of the tumor pH. This ensures that the therapeutic efficacy of the catalytic reaction is not affected by tumor acidity.

Combination with other treatment modalities: G-M synergistic therapy can be combined with other treatment modalities to enhance the overall therapeutic effect. For example, IMT can provide antigens to promote the immune system's attack on remaining tumors after apoptosis induced by catalytic reactions.

Despite these advantages, there are several challenges that need to be addressed in the development of G-M therapeutic NPs: (1) This catalytic response-based therapeutic model has been proven to be effective in the laboratory; however, the long-term toxicity caused by the catalyst is an important concern that needs to be thoroughly investigated and addressed. (2) Control of product and substrate levels: The control of product and substrate levels in each step of the catalytic reaction within the tumor is challenging and requires further research. (3) Development of NPs with human physiological characteristics: There is a need for in-depth research to develop nanoparticles that better mimic human physiological characteristics, improving their effectiveness and biocompatibility.

### Delivery designs

G-M based NPs have been designed to exploit tumor characteristics and achieve specific functions. Metal-based carriers, such as MOF and metal oxide NPs, can serve as both catalysts and carriers, overcoming the limitations of encapsulation rate for metals. Polymer and silicon-based carriers are widely recognized for their excellent safety and biocompatibility [154].

However, there are challenges associated with the utilization: (1) Most inorganic nanomaterials are difficult to degrade and eliminate from the body, leading to accumulation in organs such as the liver, lungs, and kidneys [155]. Developing inorganic NPs with good degradability is crucial to address this issue. (2) Metals used in NPs may have poor biocompatibility, necessitating appropriate chemical modifications to enhance biocompatibility and reduce toxicity. (3) The influence of loading methods on catalytic activity: The loading methods for GOx can impact its catalytic activity. Chemical conjugation and the use of organic solvents, for example, may cause damage to the protein structure of GOx and reduce its activity [156]. Thus, selecting suitable preparation technologies to minimize damage to GOx activity is important. (4) The construction methods for nanoparticles described above can be complex and lack repeatability, posing challenges for industrial-scale production. Further research and development are needed to simplify and optimize these methods.

As a result, in future research, it is important to comprehensively consider the properties of catalysts and NPs, and to design a nanodelivery system that enables the combined application of GOx and metal catalysts.

### O_2_ supply

The hypoxic condition limits the utilization of GOx, making O_2_ delivery an important aspect of ST. In many studies, NPs designed for in situ O_2_ production in tumors utilize CAT or CAT-like metal catalysts to generate O_2_ by consuming H_2_O_2_. However, this approach faces the issue of H_2_O_2_ loss, which hinders the effective activation of H_2_O_2_-responsive drugs. For instance, L-arginine requires a sufficient amount of H_2_O_2_ to release nitric oxide for gas therapy [157]. To address this challenge, exogenous O_2_ transport or alternative molecules can be employed. The following solutions can be considered:

1. External O_2_ delivery: O_2_ carriers can be categorized into perfluorocarbons and hemoglobin (Hb), both of which can be loaded with O_2_ in vitro [158, 159]. Perfluorocarbons are fully synthetic O_2_ carriers that adsorb and rapidly release O_2_ under specific conditions. Hb, a major component of blood, naturally transports O_2_ throughout the body. Hb derived from mammalian blood exhibits low immunogenicity, enabling evasion of the body’s defenses. However, these carriers have limited O_2_ carrying efficiency, and the amount of O_2_ replenished is influenced by the characteristics of the carrier.
2. In situ release of O_2_: C_3_N_4_ is a water-splitting material that can generate O_2_ by splitting water molecules under NIR light excitation [135]. Incorporating C_3_N_4_ into G-M NPs allows in situ O_2_ supplementation to enhance ST. The NPs are exposed to C_3_N_4_ in response to tumor acidity and NIR stimulation, which stimulates O_2_ production. Compared to the absence of laser stimulation, the O_2_ production rate significantly decreases. Therefore, selecting an appropriate O_2_ delivery approach is a crucial step in improving the effectiveness of synergistic therapy.

In conclusion, this review has highlighted the multiple mechanisms and advancements achievable through G-M catalytic therapy in synergistic treatment. Furthermore, the progress made in the development of nanodelivery systems for achieving synergistic therapy has been discussed. We hope that G-M, as a synergistic therapeutic agent, will provide more outstanding therapeutic strategies for cancer treatment.

## Data Availability

Not applicable.

## Abbreviations

CT: Chemotherapy
O_2_: Oxygen
GOx: Glucose oxidase
H_2_O_2_: Hydrogen peroxide
ST: Starvation therapy
CDT: Chemodynamic therapy
PDT: Photodynamic therapy
SDT: Sonodynamic therapy
PTT: Photothermal therapy
IMT: Immunotherapy
EDT: Electrodynamic therapy
CAT-like: Catalase-like
G-M: GOx-metal catalysts
NPs: Nanoparticles
PTAs: Photosensitizers
PSs: Photothermal agents
SSs: Sonosensitizers
MOFs: Metal–organic frameworks
DMSN: Dendritic mesoporous silica
US: Ultrasound
HSP: Heat shock protein
**·**OH: Hydroxyl radicals
NIR: Near-infrared light
Hb: Hemoglobin
ROS: Reactive oxygen species
Ce6: Chlorin e6
^3^O_2_: Molecular oxygen
^1^O_2_: Singlet oxygen
ICD: Immunogenic cell death
CRT: Calreticulin
GPX4: Glutathione peroxidase 4
GSH: Glutathione
DLAT: Dihydrolipoamide S-acetyltransferase
DOX: Doxorubicin
EPI: Epirubicin
3-AT: 3-Amino-1,2,4-triazole
MIL: Materials of Institute Lavoisier
MnO_2_: Manganese dioxide
ZIF: Zeolitic imidazolate framework
CaP: Calcium phosphate
CaCO_3_: Calcium carbonate
FDA: Food and Drug Administration
MnCO: Manganese carbonyl
TPZ: Tirapazamine
